# Phloem RNA-binding proteins as potential components of the long-distance RNA transport system

**DOI:** 10.3389/fpls.2013.00130

**Published:** 2013-05-10

**Authors:** Vicente Pallas, Gustavo Gómez

**Affiliations:** Instituto de Biologïa Molecular y Celular de Plantas, Consejo Superior de Investigaciones Científicas-Universidad Politécnica de ValenciaValencia, Spain

**Keywords:** RNA trafficking, RNA-binding proteins, phloem proteins, virus transport, non-coding RNAs

## Abstract

RNA-binding proteins (RBPs) govern a myriad of different essential processes in eukaryotic cells. Recent evidence reveals that apart from playing critical roles in RNA metabolism and RNA transport, RBPs perform a key function in plant adaptation to various environmental conditions. Long-distance RNA transport occurs in land plants through the phloem, a conducting tissue that integrates the wide range of signaling pathways required to regulate plant development and response to stress processes. The macromolecules in the phloem pathway vary greatly and include defense proteins, transcription factors, chaperones acting in long-distance trafficking, and RNAs (mRNAs, siRNAs, and miRNAs). How these RNA molecules translocate through the phloem is not well understood, but recent evidence indicates the presence of translocatable RBPs in the phloem, which act as potential components of long-distance RNA transport system. This review updates our knowledge on the characteristics and functions of RBPs present in the phloem.

## INTRODUCTION

RNA-binding proteins (RBPs) not only play a critical role in many aspects of post-transcriptional gene regulation (Glisovic et al., 2008), but also perform key processes of plant development, stress response, and genome organization (Fedoroff, 2002). This class of proteins contains the well-defined sequence motifs involved in RNA binding, of which the most widespread are the RNA recognition motif (RRM) and the K homology (KH) domain, which can be combined with each other or with other protein domains (Alba and Pages, 1998; Lorkovic and Barta, 2002). Other less frequent motifs are the glycine-rich motif (GRM), the double-stranded RNA binding motif (dsRBM), the Kyprides, Ouzounis, Woese (KOW) motif (acronym of the authors surname, Kyrpides et al., 1996), the coiled coil (CC) motif and the zinc finger, CCCH type motif. The *Arabidopsis* genome has been described to encode more than 200 putative RBPs, and about 250 RBP genes have been identified in *Oryza sativa* (see Ambrosone et al., 2012 for a review). Remarkably, a significant number of them have no counterpart in animals. Thus, these plant-specific RBPs are likely involved in plant-specific functions (Lorkovic, 2009), such as chloroplast mRNA stability and translation, hormone signaling and plant immunity (Woloshen et al., 2011). The phloem is a plant-specific conduit (Dinant et al., 2010; Knoblauch and Oparka, 2012; Zhang et al., 2012) that delivers not only sugars, amino acids, mineral nutrients and hormones, but also peptides, proteins and RNAs from autotrophic to heterotrophic tissues (Lough and Lucas, 2006; Dinant and Lemoine, 2010; Atkins et al., 2011). After demonstrating that RNA movement possibly lies behind a range of long-distance signals in plants (Wu et al., 2002), RNA emerged as a non-cell autonomous information macromolecule (Lucas et al., 2001). Different types of RNA molecules are transported through the phloem, including the mRNAs, viral RNAs (vRNAs) and small RNAs (sRNAs) deriving from RNA silencing machinery (Kehr and Buhtz, 2008). The long-distance transport of certain host non-cell autonomously acting RNAs has been shown to be a key regulator of essential processes such as gene silencing, pathogen defense, development, stress response, and parasitic interactions (Kim et al., 2001; Yoo et al., 2004; Haywood et al., 2005; Banerjee et al., 2009; Dunoyer et al., 2010; Melnyk et al., 2011; LeBlanc et al., 2012; Kang et al., 2013). There are several mRNAs for which the mobile nature and their systemic activity has been clearly demonstrated. Among these, the best examples known are the RNA of *GA INSENSITIVE* (*GAI*) involved in leaf development (Haywood et al., 2005), the tomato *mouse ears* (Me) transcript (Kim et al., 2001) responsible of a characteristic leaf shape, the *StBEL5* mRNA that, regulated by the untranslated regions (UTRs), its vascular movement correlates with enhanced tuber production (Banerjee et al., 2009; Cho et al., 2012) and the *Arabidopsis FLOWERING LOCUS T* that contributes to systemic floral signaling (Lu et al., 2012). Recently, phloem-mobile *Aux/IAA* transcripts have been shown to be target to the root tip and modify root architecture (Notaguchi et al., 2012). How these mRNAs, vRNAs, and sRNAs are transported through the phloem is still an open question. However, it is commonly accepted that without the protection of specific RBPs, naked, cellular RNA molecules quickly fall prey to the degradative process (Shyu et al., 2008). Although no RNase activity has been reported in sieve tube exudates, which suggests that there might be limited RNA degradation in the sieve tubes, the presence of 3′–5′ exoribonuclease in the phloem of several species has been described (e.g., Lin et al., 2009). Initial progress was made in advancing in the knowledge of RNA trafficking in plants thanks to studies dealing with inter-cellular and vascular movements of plant viruses. Indeed, the first phloem RBP (the pumpkin PHLOEM PROTEIN16, CmPP16) was identified on the basis of its cross-reactivity with a viral movement protein and was demonstrated to be a functional homolog of these protein classes (Xoconostle-Cazares et al., 1999). The similarity between the mechanisms that plant viruses use to move systemically and RNA transport in the phloem was immediately suggested (Thompson and Schulz, 1999; Lucas et al., 2001). Increasingly more RBPs in the phloem of different plant species have been detected and some have been characterized in detail. Here, we update current knowledge on the main phloem RBPs described to date for which a potential function in long-distance RNA transport has been suggested and/or demonstrated.

## THE *Cucurbita maxima* PHLOEM PROTEIN 16

Increasing evidence for diverse plant-mRNA molecules being detected in vascular tissue has led to speculate that endogenous RNAs can traffic through companion cell-sieve element (CC-SE) plasmodesmata similarly, to the way in which plant viruses establish systemic infection. This conceptual framework has not only promoted the search for phloem-specific proteins whose functions can parallel those of plant viral movement proteins (MPs), but has also enabled the identification of the 16-kD *Cucurbita maxima* phloem protein (CmPP16), a RBP with immunological cross-reactivity and a functional similarity to the MP of the *Red clover necrotic mosaic virus* (RCNMV; Xoconostle-Cazares et al., 1999). R-CmPP16-1 was seen to bind both sense and antisense *CmPP16-1 *RNA, as well as RCNMV RNA2, but failed to interact with both single-stranded and double-stranded DNA. In this pioneer work, microinjection experiments followed by *in situ* localization helped determine that CmPP16 is able to interact with plasmodesmata to induce an increase in the size-exclusion limit and to potentiate its own cell-to-cell transport. Heterografting assays were used to demonstrate that both CmPP16 mRNA and the protein are able to move from pumpkin stock CC into the SE through plasmodesmata, and that they can be detected specifically in the cucumber scion. This study provided the first evidence that an RNA-binding phloem protein (CmPP16) can mediate RNA transport through plasmodesmata into the phloem translocation stream by playing a relevant role in the non-cell autonomous regulation of gene expression in plants.

A more recent study (Aoki et al., 2005), which used a heterologs system that permits this pumpkin phloem protein to be expressed in rice (*Oryza sativa*), showed that the shoot-ward translocation of CmPP16 appears to be passively carried by the bulk flow, whereas the root-ward traffic of this RBP is selectively controlled. Furthermore, it has been demonstrated that CmPP16 can form stable complexes with both the previously described phloem factors, eukaryotic translation initiation factor 5A (eIF5A) and the translationally controlled tumor-associated protein (TCTP; see below). According to these results, the authors proposed the existence of a destination-selective long-distance trafficking mechanism, potentially regulated by protein–protein interactions, which can mediate the targeting of endogenous RNAs selectively to the shoot and/or to the root. Two models for how the destination of CmPP16 movement is determined were proposed by the authors (Aoki et al., 2005): (i) a lateral transfer of CmPP16 proteins between sieve tubes could be controlled selectively at the nodal region where vascular bundles are cross-connected as occurs for the distribution of photosynthate or that (ii) destination is determined by selective exit from the sieve tube system as reported for some RNAs, specific phloem proteins, viral movement proteins, and small solutes.

## PHLOEM LECTINS: PHLOEM PROTEIN 2 AND PHLOEM LECTIN 17

**FIGURE 1:**
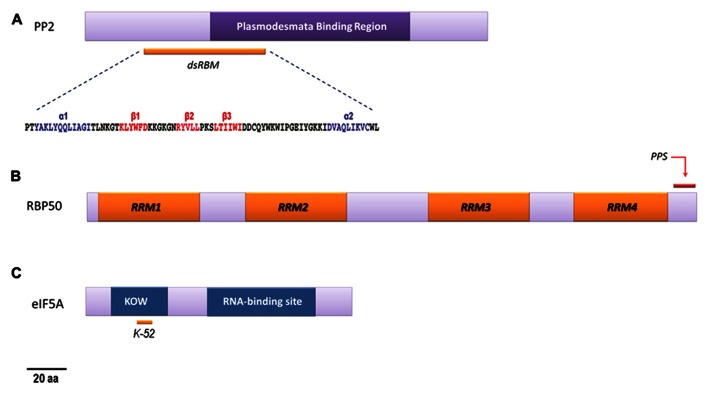
**Schematic representation of RNA-binding phloem proteins.** **(A)** PP2: the position of the conserved region, proposed to be involved in specific plasmodesmata recognition (Balachandran et al., 1997), is indicated. The motif (*dsRBM*) predicted to be responsible of its RNA-binding activity is also shown. For the potential dsRBM sequence, helices α1 and α2 are shown in blue, the β1, β2, and β3 sheets are represented in red, and coils are shown in gray (modified from Gómez and Pallás, 2004). **(B)** CmRBP50: the diagram indicates the location of the four conserved RNA recognition motifs (RRM1–RRM4) that are essential for the RNA-binding properties. The C-terminus region is highly rich in putative phosphorylation sites (PPS), demonstrated to be critical for RNP complex assembly, as shown (modified from Li et al., 2011). **(C)** eIF5A: graphic representation containing both predicted KOW (Kyrpides et al., 1996) and eIF5A RNA-binding motifs. kow motif is thought to contain the hypusination site (Lysine 52′ in CmelIF5A- isoforms, marked).

Phloem protein 2 from cucumber (CsPP2), a protein whose apparent molecular mass comes close to 26 kDa, was the first endogenous phloem-factor reported to bind viroid RNAs *in vitro *(Gómez and Pallás, 2001; Owens et al., 2001). The lectins belonging to the PP2 family are among the most abundant and enigmatic proteins in phloem sap. Moreover the phylogeny analyses identified PP2-like genes in species from 17 angiosperm and gymnosperm genera, and indicated that these proteins are ancient and common in vascular plants. Despite the differences in size and amino acid sequences, it was reported that all these proteins maintain cell-specific gene expression, overall domain structure, and lectin activity (Dinant et al., 2003). Although protein accumulates in SEs, *in situ* hybridisation experiments showed that CmPP2 mRNA is detected only in CCs (Bostwick et al., 1992). Consistently, the corresponding promoter sequence drove the GUS expression predominantly in phloem tissues, although specificity was lower than that of the specific-phloem promoters of a viral origin (Guo et al., 2004). In *Arabidopsis*, the closest ortholog of phloem lectin PP2, PP2-A1, was encoded by a gene specifically expressed in the phloem (Dinant et al., 2003) and accumulated mostly in the SEs (Batailler et al., 2012). Interestingly, the PP2 of the *Cucurbitaceae *spp. has been shown to have the capacity to interact with and to modify the size-exclusion limit of plasmodesmata (**Figure 1**) and to move from cell-to-cell when microinjected into mesophyll cells of cucurbit cotyledons (Balachandran et al., 1997). In addition, PP2 of *Cucurbita maxima *has been seen to move from CCs, where it is synthesized, into sieve tubes (Golecki et al., 1999) to translocate in the intergeneric grafts in the assimilate stream toward sink tissues (Fisher et al., 1992; Golecki et al., 1999). All together, these biochemical characteristics support the hypothesis that members of this phloem protein family might be involved in the long-distance movement of endogenous and/or exogenous RNAs in plants. Indeed, immunoprecipitation experiments have revealed that CsPP2 is capable of interacting *in vivo* with a viroid RNA, while intergeneric graft assays have shown that both CsPP2 and viroid RNA translocate to the scion. The translocated viroid was symptomatic in the non-host scion, indicating that translocated RNA is functional. A dsRBM, which was identified in the predicted structure of CsPP2, was proposed to be responsible for the RNA-binding properties of this cucumber phloem protein (**Figure 1**; Gómez and Pallás, 2004). Viroids are plant-pathogenic non-protein-coding RNAs, thus all the critical steps of their biological cycle must be assisted by host factors (reviewed in Flores et al., 2005; Ding, 2009; Gómez and Pallás, 2013). The long-distance movement of viroids occurs, as in most plant viruses, by translocation via the phloem (Zhong and Ding, 2008). Consequently, it is reasonable to assume that phloem protein(s) can interact with viroid RNA to assist its translocation to distal plant regions. The matter of whether this type of proteins forms part of endogenous ribonucleoprotein (RNP) complexes to translocate host mRNAs remains controversial. The RNA binding of CsPP2 seems to be sequence non-specific (Gómez and Pallás, 2004). In fact, 75 different transcripts have been cloned from PP2 complexes after co-IP experiments using PP2 antisera (Ham et al., 2009), revealing that this protein binds to a broad spectrum of mRNA species.

In a more recent work, Bencharki et al. (2010) reported that the protein members of the PP2 family in cucumber interact with the *Cucurbit aphid borne yellows virus *(CABYV), can stimulate virus transmission by aphid and that they possess a protective activity against virion degradation *in vitro*.

Another phloem lectin showing RNA-binding capability has been identified in exudates from melon plants (*Cucumis melo*; Gómez et al., 2005). This phloem protein, of 17 kDa (CmmLec17), binds to different pathogenic and non-pathogenic RNAs, and accumulates in melon phloem sap. CmmLec17 is homologous to melon PP2 (CmmPP2), and the most obvious difference between both proteins is that 62 amino acids are lacking in the CmmLec17 N-terminus. Unexpectedly, no RRM is evident from its primary structure. The central region, proposed to contain the essential motif(s) for the interaction with plasmodesmata receptors and to also increase their size-exclusion limits in PP2 (Balachandran et al., 1997), is highly conserved in Cmmlec17 (Dinant et al., 2003), which explains their potential to be exported to phloem traffic. Intergeneric graft assays have demonstrated that Cmm-Lec17 is a translocatable phloem protein capable of moving from melon stocks to pumpkin scions. The fact that CmmLec17 mRNA is absent in phloem exudates dismisses the possibility that CmmLec17 precursors could be exported from CCs to SEs and thus, that the protein could be synthesized *de novo* in scion tissues.

## *Cucurbita maxima* PHLOEM SMALL RNA BINDING PROTEIN1

The biochemical analysis of pumpkin (*Cucurbita maxima*) phloem sap led Yoo et al. (2004) to identify a protein of 21 kDa capable of preferentially binding to small single-stranded RNAs (ssRNAs; with a 1,000-fold higher affinity than dssRNAs). The protein presented two highly repetitive sequences, HGHGPA(S)GG and CQPANPNVGH, 16 imperfect octapeptide (P-A/S-G/W-G-H-G-H/C-G) repeats, and three decapeptide repeats (H-C-Q-P-A-N/S-P-N-V-G), but no highly homologous genes. The putative PSRP1 orthologs in cucumber and lupin displayed similar binding specificity, except that this phloem protein did not appear to bind small dsRNA in lupin. Interestingly, microinjection experiments have revealed that PSRP1 can mediate the cell-to-cell trafficking of 25 nucleotide single-stranded, but not double-stranded, RNA molecules, which led the authors to propose PSRP1 as a *bona fide* candidate to be involved in systemic si/miRNA transport. However, there is not genetic evidence to support the function of this protein. In addition, Dunoyer et al. (2010) recently demonstrated that siRNA duplexes, as opposed to single strands, are likely to be the silencing signals between plant cells, and possibly over long-distances, thus dismissing the possibility of PSRP1 being the protein responsible for translocating the mobile silencing signal.

## PUMPKIN EUKARYOTIC TRANSLATION INITIATION FACTOR 5A

Eukaryotic translation initiation factor 5A is a small, single polypeptide protein (~18 kDa) found in all eukaryotic kingdoms (Gordon et al., 1987; Park et al., 1988; Hu et al., 2005). Originally, it was deemed to play a role in translation initiation (Kemper et al., 1976; Benne and Hershey, 1978), although recent results indicate that it may participate in ribosome-related events, such as protein folding, rather than polypeptide chains synthesis (Jao and Chen, 2006; Zanelli et al., 2006). The capacity of eIF5A to bind mRNA has been confirmed by Xu and Chen (2001), who also identified, using SELEX (systematic evolution of ligands by exponential enrichment), the preferential sequence to which eIF5A binds (consensus sequence AAAUGUCACAC) and the transcripts bound by human eIF5A (Xu et al., 2004). Recently, eIF5A was demonstrated to interact with the 3′UTR of the phloem-mobile *StBEL5* mRNA (Cho et al., 2012) suggesting its potential participation as a component of the ribonucleotide complexes that function to regulate RNA transport or metabolism. eIF5A contains a KOW motif (acronym of the authors surname, Kyrpides et al., 1996) characterized by the presence of an invariant glycine residue and five alternating blocks of hydrophilic and hydrophobic residues (**Figure 1**). In addition, at the C-terminal region the protein contains the eIF5A motifs for ribosome binding, RNA-binding and translation activity. This protein is considered as a bimodular protein, capable of interacting with protein and nucleic acid being the KOW motif required for both type of interactions. The RNA-binding capacity was shown to be dependent on hypusination (lysine residue modification) of the protein (Xu and Chen, 2001). The hypusinable lysine would be contained in the KOW motif (**Figure 1**). Several isoforms have been described in different plant species. In *Arabidopsis*, three isoforms (eIF5A1, eIF5A2, and eIF5A3) have been described with distinct expression patterns and apparent different functions. AteIF5A1 is localized in senescing leaf tissue suggesting that it plays a role in senescence (Duguay et al., 2007). AteIF5A2 has been shown to be involved in both pathogen-induced cell death and the development of disease symptoms (Hopkins et al., 2008). The *Arabidopsis* eIF5A3 isoform has been demonstrated to influence growth and responses to osmotic and nutrient stress, and has been detected in the phloem of roots, stems, and leaves, particularly CCs (Ma et al., 2010a). Pumpkin eIF5A (CmeIF5A) has been isolated from phloem sap as an interaction partner with CmPP16 by a combination of gel-filtration chromatography and co-immunoprecipitation experiments (Aoki et al., 2005) and as a component of the RBP50-based RNP complex (Ham et al., 2009). Ma et al. (2010b) have demonstrated its RNA binding capability to different mRNAs, although at a lower affinity for RNA as compared with CmPP16. In addition, heterografting experiments have revealed that some CmeIF5A isoforms can move long-distances through the phloem and that the hypusination of CmeIF5A is not essential for it to enter the sieve tube system (Ma et al., 2010b). The transcripts for all four CmeIF5A isoforms are able to enter the sieve tube system, whereas only transcripts CmeIF5A2 and CmeIF5A4 can move across the graft union from the pumpkin stock to the cucumber scion phloem system. Since all four CmeIF5A transcripts and the encoded proteins were identified in the pumpkin phloem sap, this raises the question as to why both transcripts and proteins would traffic from CCs into the SEs. As stated by the authors (Ma et al., 2010b) it is possible that the four CmeIF5A transcripts may be delivered to sink tissues where translation could take place. Alternatively, translation of these transcripts may occur in the sieve tube system from whence they could function locally or act as long-distance signals.

Recently, eIF5A was reported to be induced in tomato plants infected with *Citrus exocortis viroid* (CEVd), although no direct interaction with this RNA was detected (Lison et al., 2013).

## RNA BINDING PROTEIN 50

Pumpkin RBP50, a polypyrimidine tract-binding (PTB) protein, has been identified to be the core of a RNP complex present in the phloem sap of pumpkin plants (Ham et al., 2009). In *Arabidopsis*, three genes encoding proteins homologous to mammalian PTBs have been identified. Two of these *Arabidopsis* PTB homologues have been suggested to be involved in pollen germination (Wang and Okamoto, 2009). Recently, Rühl et al. (2012) demonstrated that these proteins can control alternative splicing and the expression of fundamental flowering regulator genes. A series of grafting experiments and gel mobility shift assays using three previously identified phloem-mobile mRNAs led Ham et al. (2009) to show that RBP50 is a phloem-mobile RBP that may exhibit selective binding to the population of transcripts (it binds GAIP and PP16-1 transcripts, but it does not appear to interact with the RNA for RINGP) entering the translocation stream. RBP50 has four RRM with several Ser residues that can be potentially phosphorylated (**Figure 1**). Li et al. (2011) showed that phosphoserine residues, located at the C-terminus of CmRBP50, are critical for RNP complex assembly. Several proteins have been identified as potential components of the RBP50-based phloem RNP complex, including eIF5A. Since, as stated above, Aoki et al. (2005) reported that CmPP16 can interact with eIF-5A, it is possible that the proposed RBP50- eIF-5A is not direct. Remarkably, the heterografting studies and co-IP experiments performed on phloem sap collected from pumpkin stock, cucumber scion or ungrafted cucumber plants revealed that specific phloem mRNAs are carried through the phloem translocation stream within RBP50 RNP complexes. This set of experiments sophisticatedly demonstrated the existence of a CmRBP50-based RNP complex which is able to transport a specific set of mRNAs, including those encoding transcription factors, from mature leaves to developing tissues/ organs.

## CONCLUDING REMARKS

Considerable progress has been made in the knowledge of the RNA and protein composition of angiosperm phloem. Of the large number of these identified macromolecules, a significant number has been shown to perform a non-cell autonomous function, mainly in relation to defense and developmental processes. Almost 100 non-redundant RNA-RBPs have been detected in the phloem sap of different plant species (Yoo et al., 2004; Gómez et al., 2005; Giavalisco et al., 2006; Aki et al., 2008; Lin et al., 2009), but their functions remain to be elucidated. Although we now have a reasonable idea of how some host transcripts and vRNAs can be transported through the phloem, the way the silencing mobile signal reaches distal plant parts is still a mystery. Genetic approaches have successfully revealed the mobile nature of the silencing signal (Dunoyer et al., 2010; Molnar et al., 2010), but have failed to elucidate its physical form, probably because factors involved in its movement are essential for plant development. Thus, biochemical approaches such as the yeast three-hybrid system can be very convenient alternatives to try to identify proteins that interact with phloem-mobile RNAs (Cho et al., 2012).

## Conflict of Interest Statement

The authors declare that the research was conducted in the absence of any commercial or financial relationships that could be construed as a potential conflict of interest.
